# Supplementary material for: Ultrasound Assisted Synthesis and In Silico Modelling of 1,2,4-Triazole Coupled Acetamide Derivatives of 2-(4-Isobutylphenyl)propanoic acid as Potential Anticancer Agents
Source: Molecules. 2022 Nov 17;27(22):7984. doi: 10.3390/molecules27227984 (PMC9698963; doi:10.3390/molecules27227984)
Supplement: Supplementary file 1 [file molecules-27-07984-s001.zip › molecules-1950360-supplementary.pdf]

Supplementary Data

Article

"Ultrasound Assisted Synthesis and *In Silico* Modelling of 1,2,4-triazole Coupled Acetamide Derivatives of 2-(4-isobutylphenyl) propanoic acid as Potential Anticancer Agents"

Sadaf Mahmood [1], Samreen Gul Khan[1,*], Azhar Rasul [2], Jørn Bolstad Christensen [3], Mohammed A.S Abourehab[4,*]

[1] Department of Chemistry, Drug Design and Medicinal Chemistry Laboratory, Faculty of Physical Science, Government College University, Faisalabad-38000, Pakistan

[2] Department of Zoology, Faculty of Life Sciences, Government College University Faisalabad, 38000 Faisalabad, Pakistan.

[3] Department of Chemistry, Faculty of Science, University of Copenhagen, Copenhagen Denmark.

[4] Department of Pharmaceutics College of Pharmacy, Umm Al-Qura University, Makkah 21955, Saudi Arabia

* Correspondence: samreengul@gcuf.edu (S.G.K.); maabourehab@uqu.edu.sa (M.A.S.A.);

Tel.: +92-300-427-0077 (S.G.K.).

Table of Contents:

Figure S1: ^{1}H NMR spectrum of compound **2** (Full spectrum) 3
Figure S2: ^{1}H NMR spectrum of compound **3** (Full spectrum) 3
Figure S3: ^{1}H NMR spectrum of compound **4**(Full spectrum) 4
Figure S4: ^{1}H NMR spectrum of compound **4** (aromatic region) 4
Figure S5: ^{1}H NMR spectrum of compound **4** (aliphatic region) 5
Figure S6: ^{13}C NMR spectrum of compound **2** 5
Figure S7: ^{13}C NMR spectrum of compound **3** 6
Figure S8: ^{13}C NMR spectrum of compound **4** 6
Figure S9: ^{1}H NMR spectrum of compound **6a** (Full) 7
Figure S10: ^{1}H NMR spectrum of compound **6a** (aromatic region) 7
Figure S11: ^{1}H NMR spectrum of compound **6a** (aliphatic region) 8
Figure S12: ^{13}C NMR spectrum of compound **6a** (Full spectrum) 8
Figure S13: ^{1}H NMR spectrum of compound **6b** (Full spectrum) 9
Figure S14: COSY- ^{1}H NMR spectrum of compound **6b** 9
Figure S15: ^{13}C NMR spectrum of compound **6b** (Full spectrum) 10
Figure S16: ^{1}H NMR spectrum of compound **6c** (Full spectrum) 10
Figure S17: ^{1}H NMR spectrum of compound **6c** (aromatic region) 11
Figure S18: COSY- ^{1}H NMR spectrum of compound **6c** 11
Figure S19: ^{13}C NMR spectrum of compound **6c** (Full spectrum) 12
Figure S20: ^{1}H NMR spectrum of compound **6d** (Full spectrum) 12
Figure S21: COSY ^{1}H NMR spectrum of compound **6d** 13
Figure S22: ^{13}C NMR spectrum of compound **6d** (Full spectrum) 13
Figure S23: ^{1}H NMR spectrum of compound **6e** (Full) 14
Figure S24: COSY ^{1}H NMR spectrum of compound **6e** 14
Figure S25: ^{13}C NMR spectrum of compound **6e** (Full spectrum) 15
Figure S26: ^{1}H NMR spectrum of compound **6f** (Full Spectrum) 15
Figure S27: COSY ^{1}H NMR spectrum of compound **6f** 16
Figure S28: ^{13}C NMR spectrum of compound **6f** 16
Figure S29: Mass spectrum of compound **6a** 17
Figure S30: Mass spectrum of compound **6b** 18
Figure S31: Mass spectrum of compound **6c** 19
Figure S32: Mas spectrum of compound **6d** 20
Figure S33: Mass spectrum of compound **6e** 21
Figure S34: Mass spectrum of compound **6f** 22

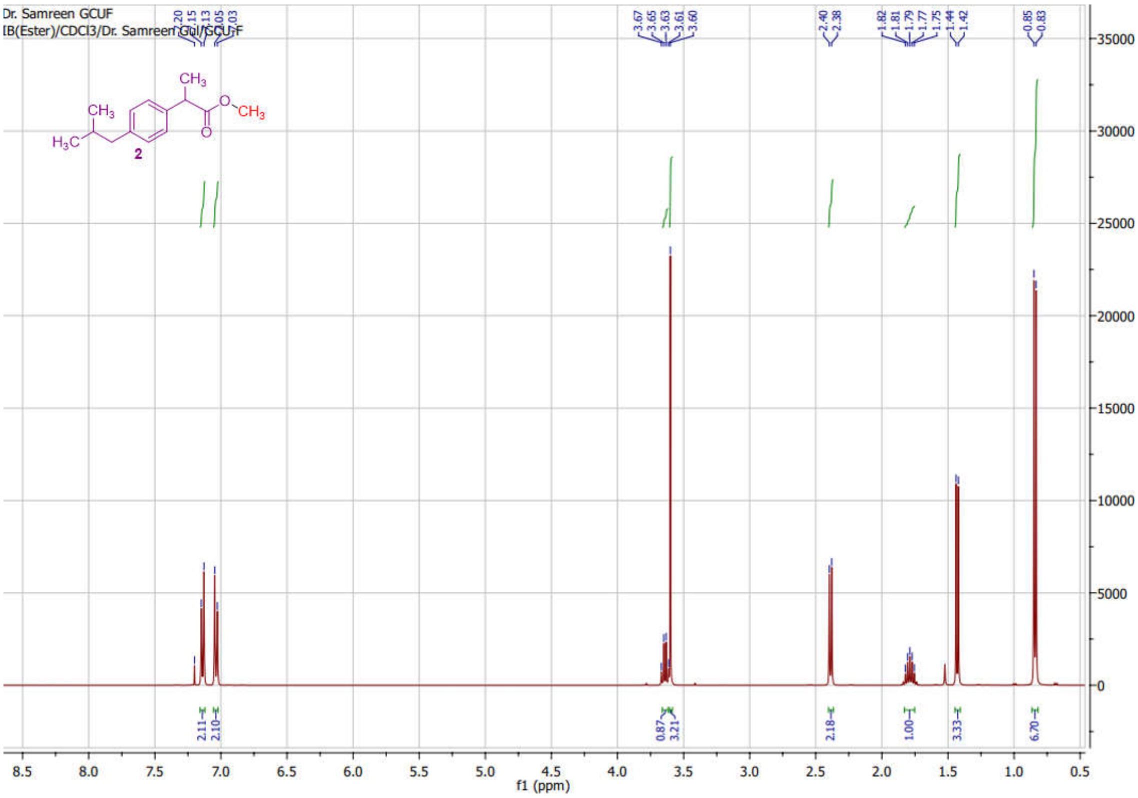

Figure S1: ^{1}H NMR spectrum of compound **2** (Full)

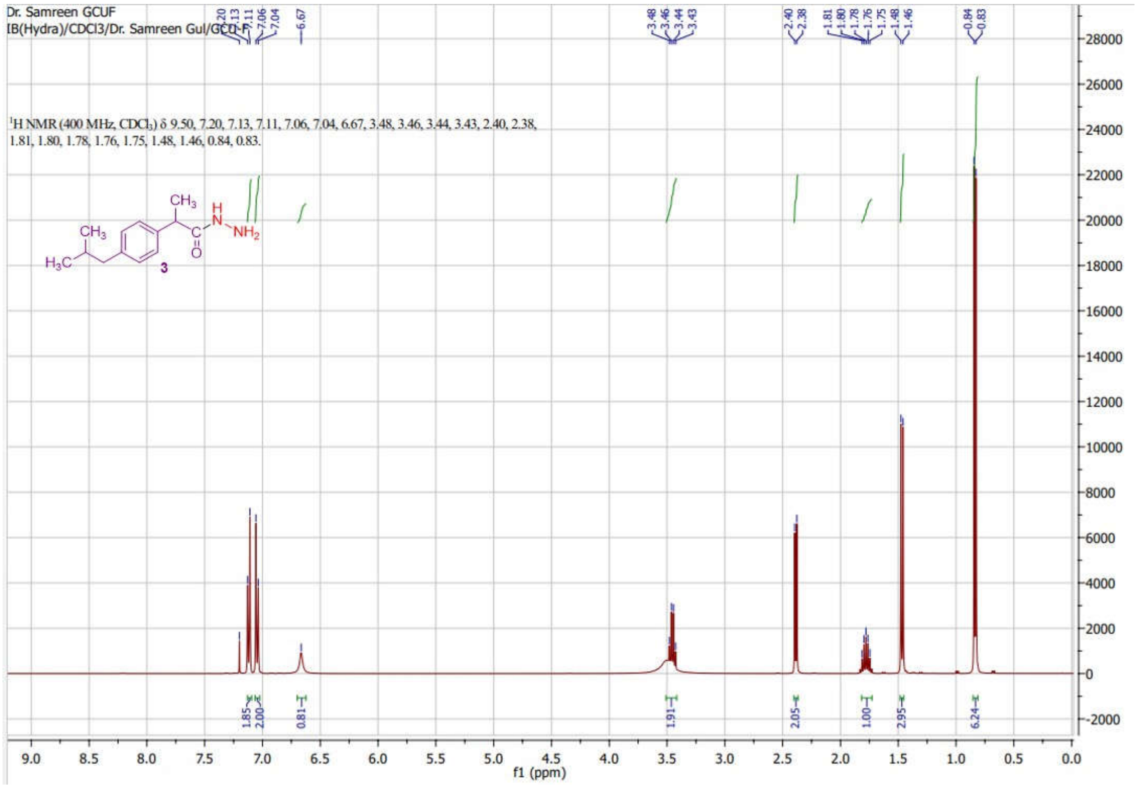

Figure S2: ^{1}H NMR spectrum of compound **3** (Full)

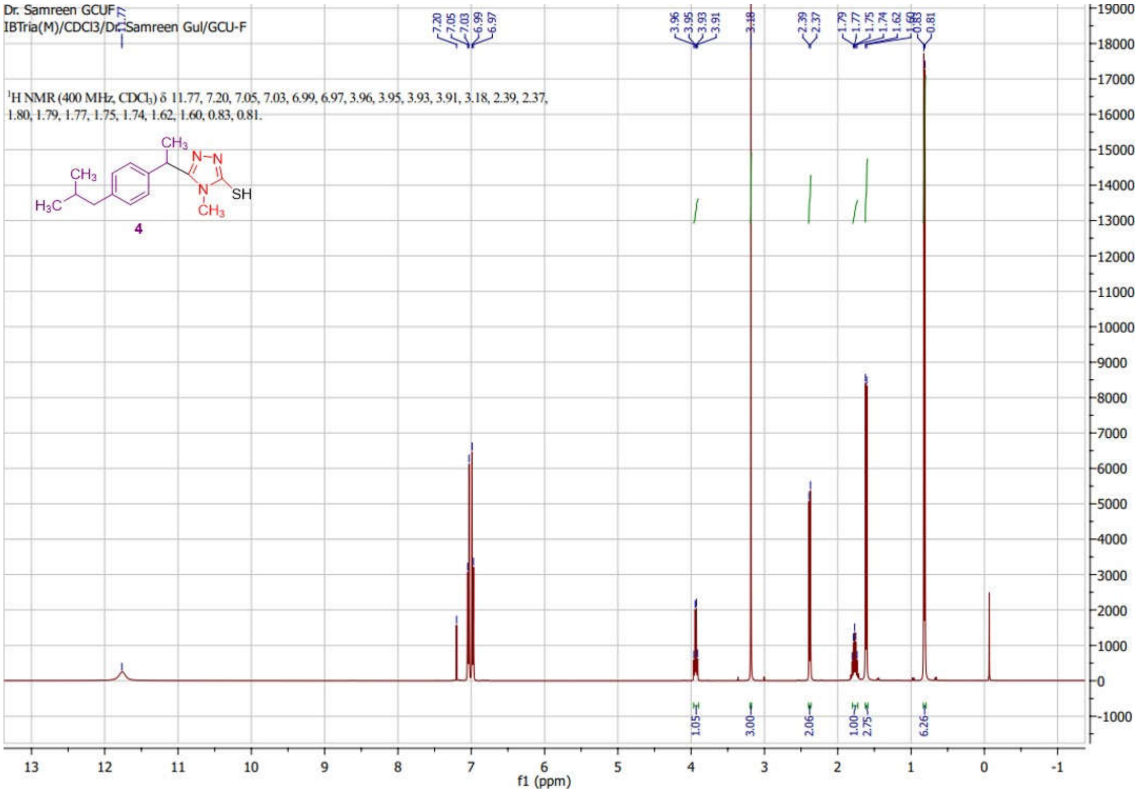

Figure S3: ^{1}H NMR spectrum of compound **4**(Full)

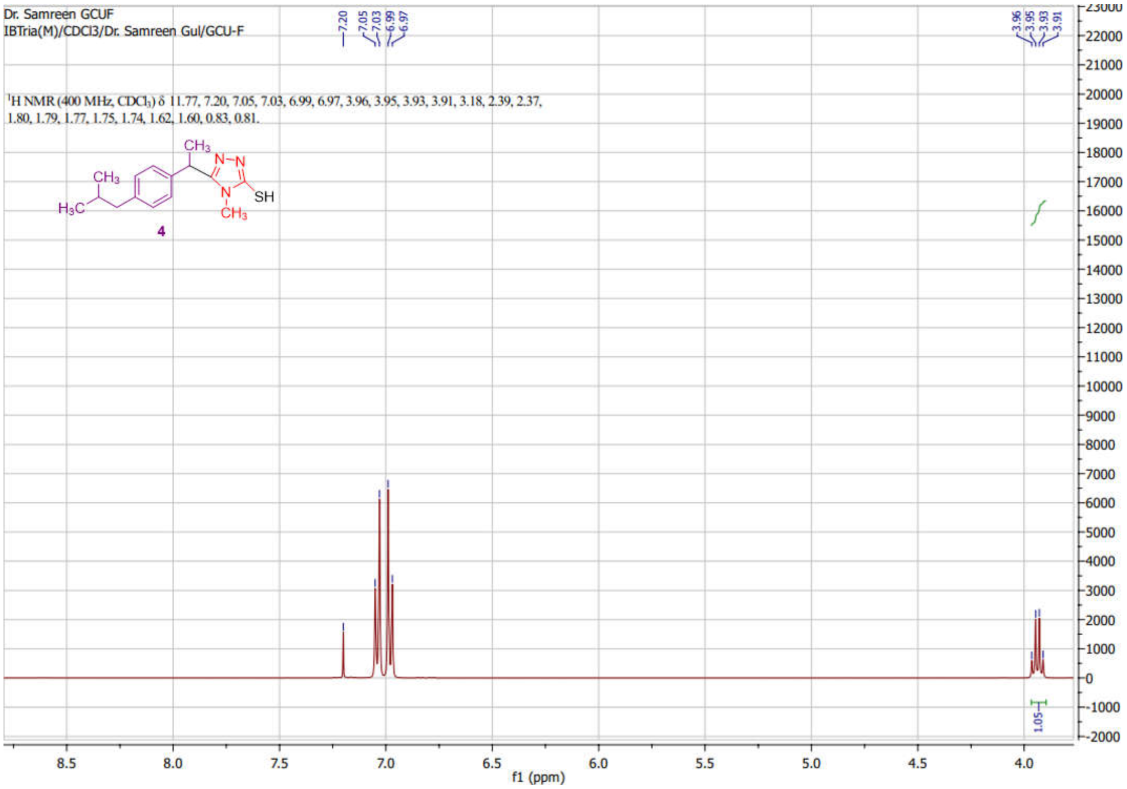

Figure S4. ^{1}H NMR spectrum of compound **4** (aromatic region)

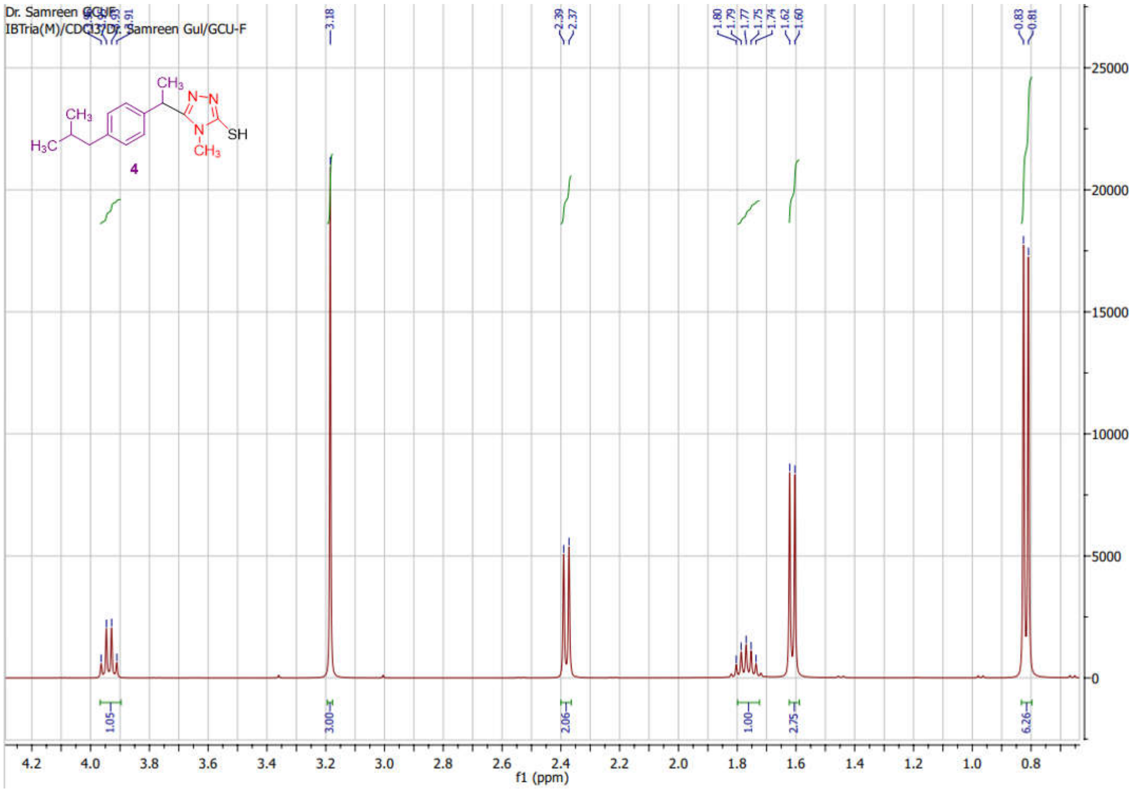

Figure S5. ^{1}H NMR spectrum of compound **4** (aliphatic region)

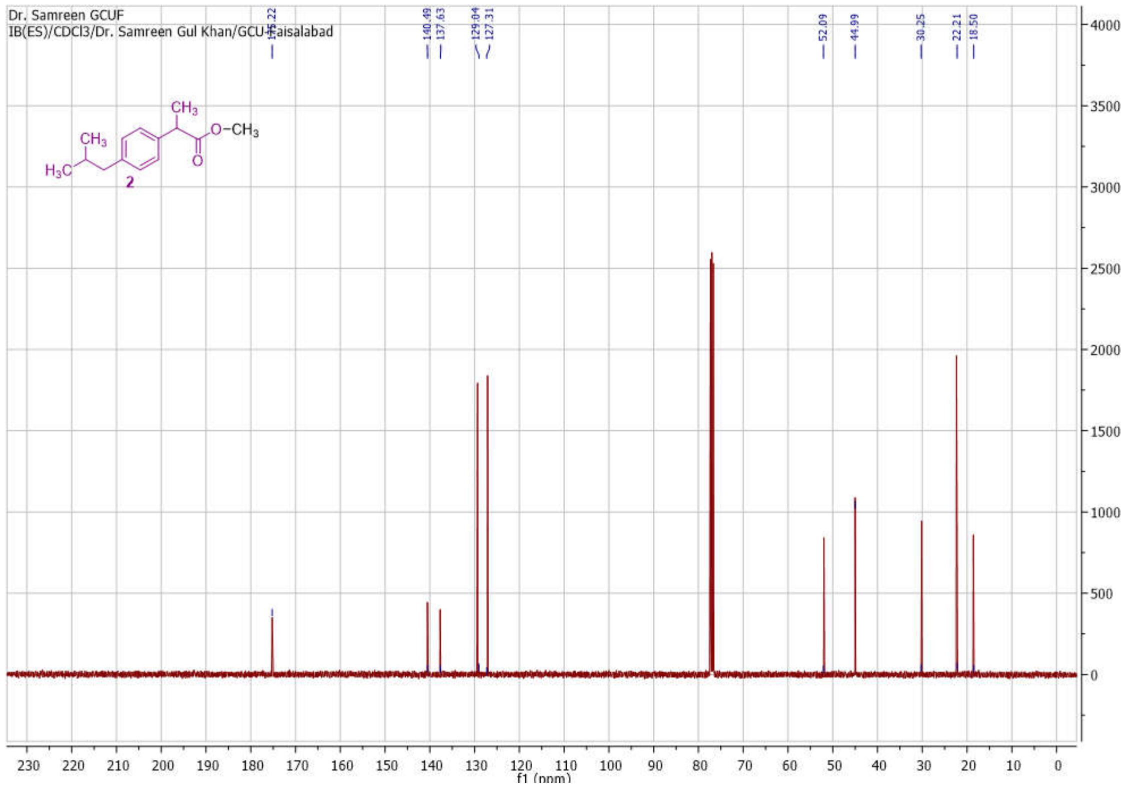

Figure S6. ^{13}C NMR spectrum of compound **2**

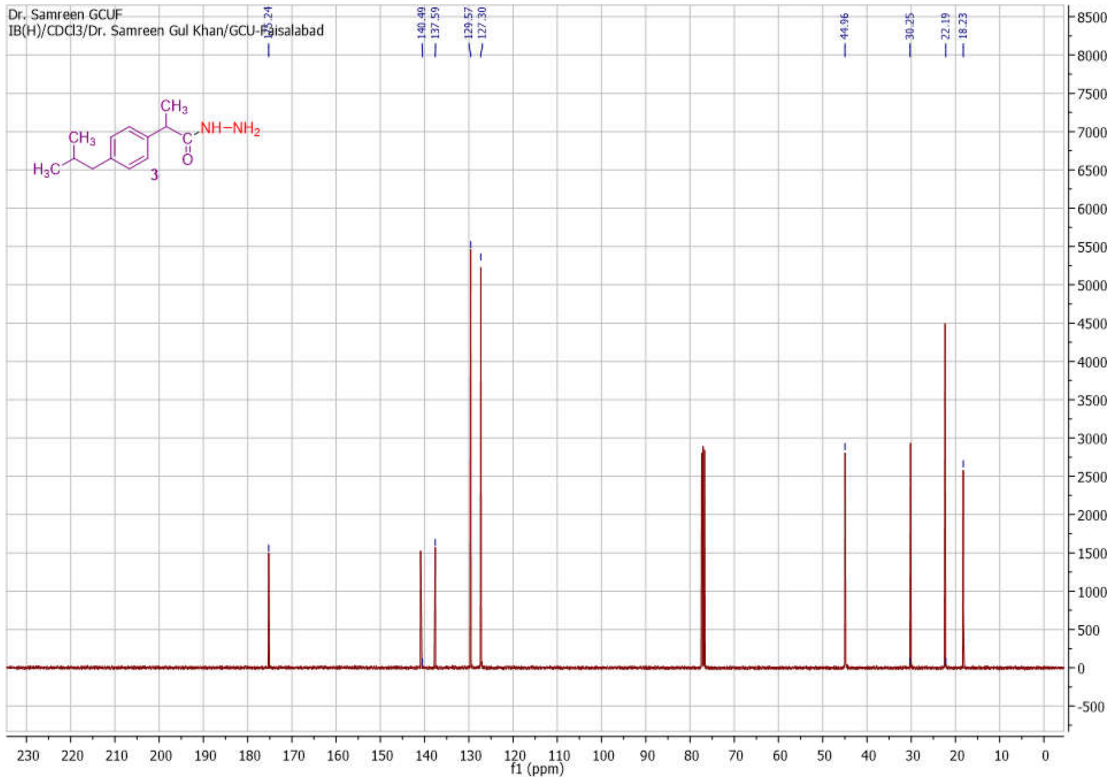

Figure S7. ^{13}C NMR spectrum of compound **3**

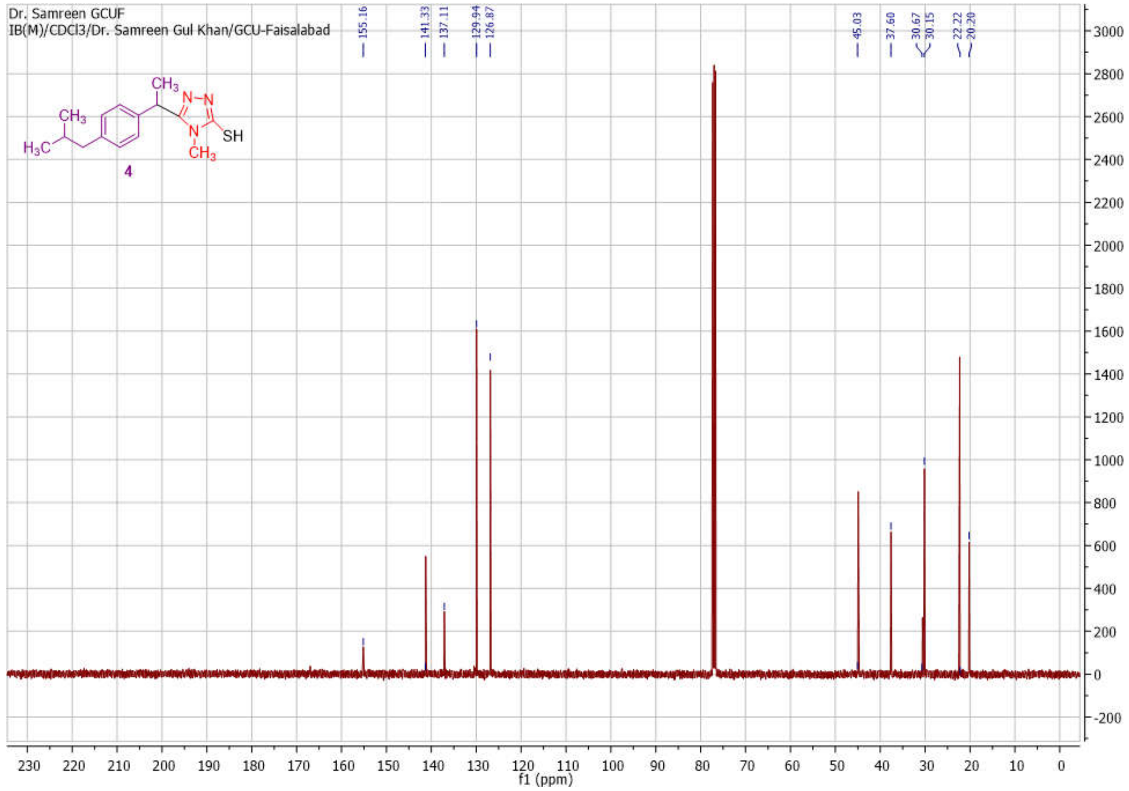

Figure S8. ^{13}C NMR spectrum of compound **4**

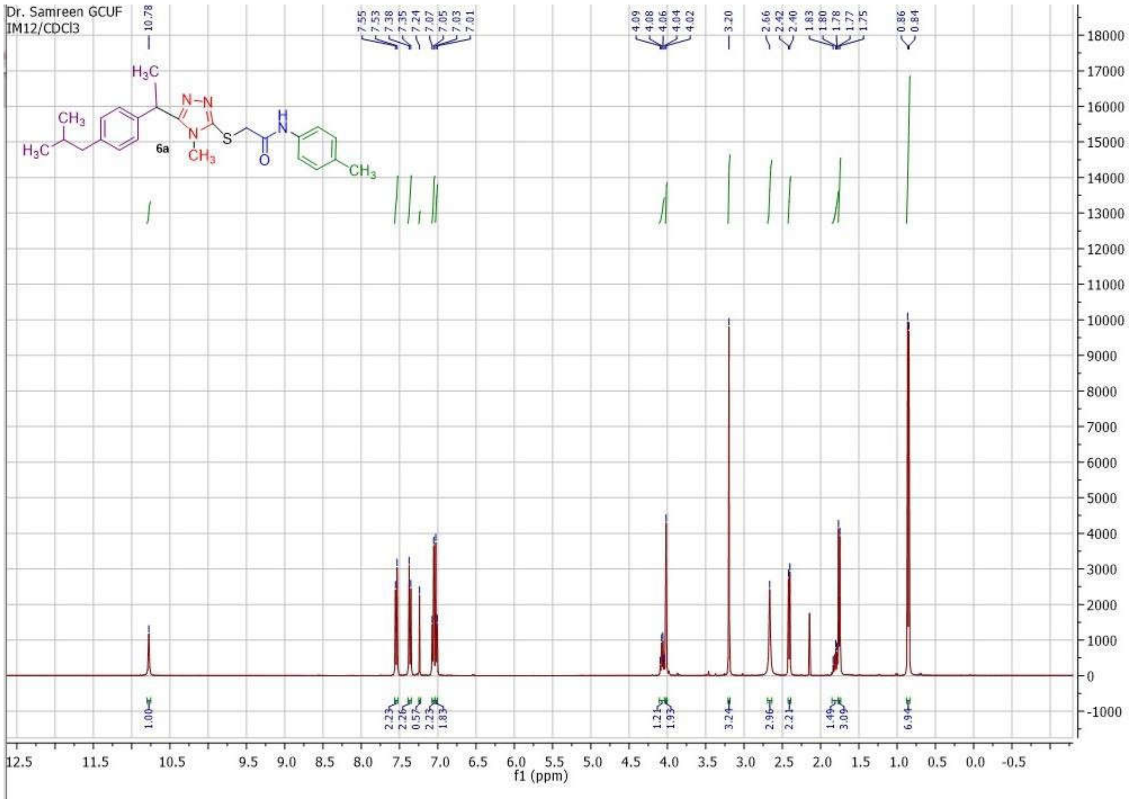

Figure S9: ^{1}H NMR spectrum of compound **6a** (Full)

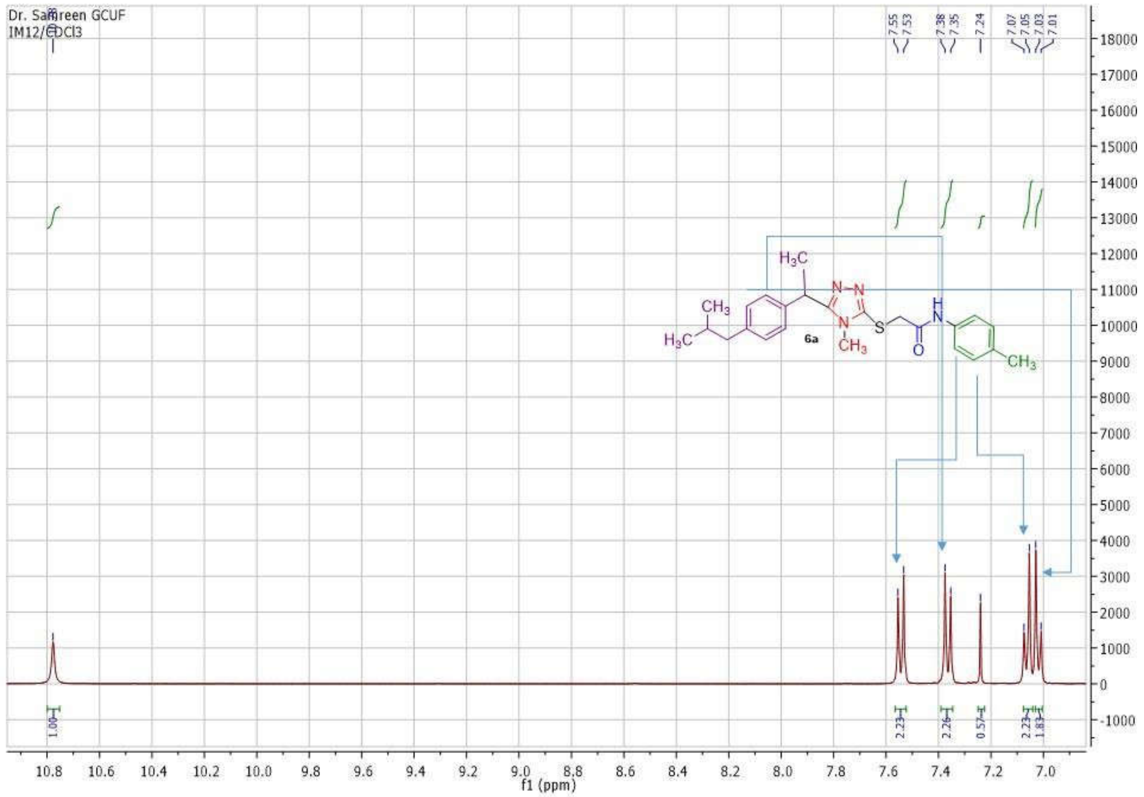

Figure S10. ^{1}H NMR spectrum of compound **6a** (aromatic region)

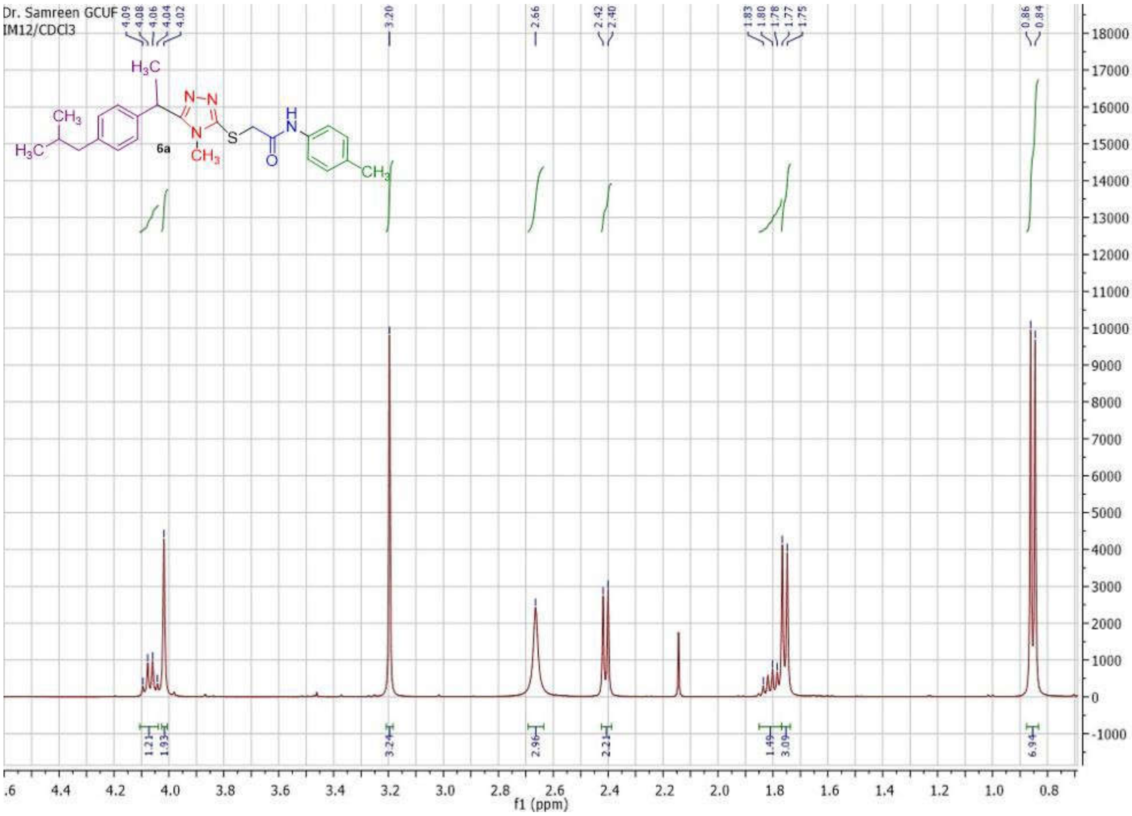

Figure S11. ^{1}H NMR spectrum of compound **6a** (aliphatic region)

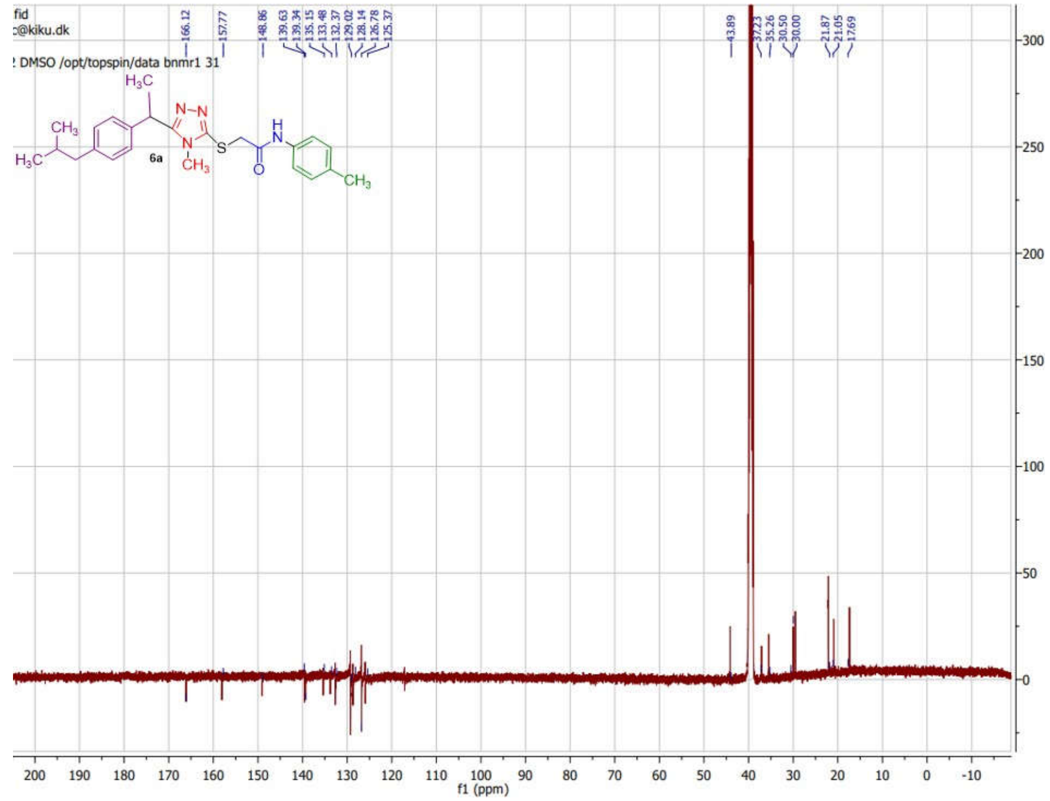

Figure S12. ^{13}C NMR spectrum of compound **6a** (Full spectrum)

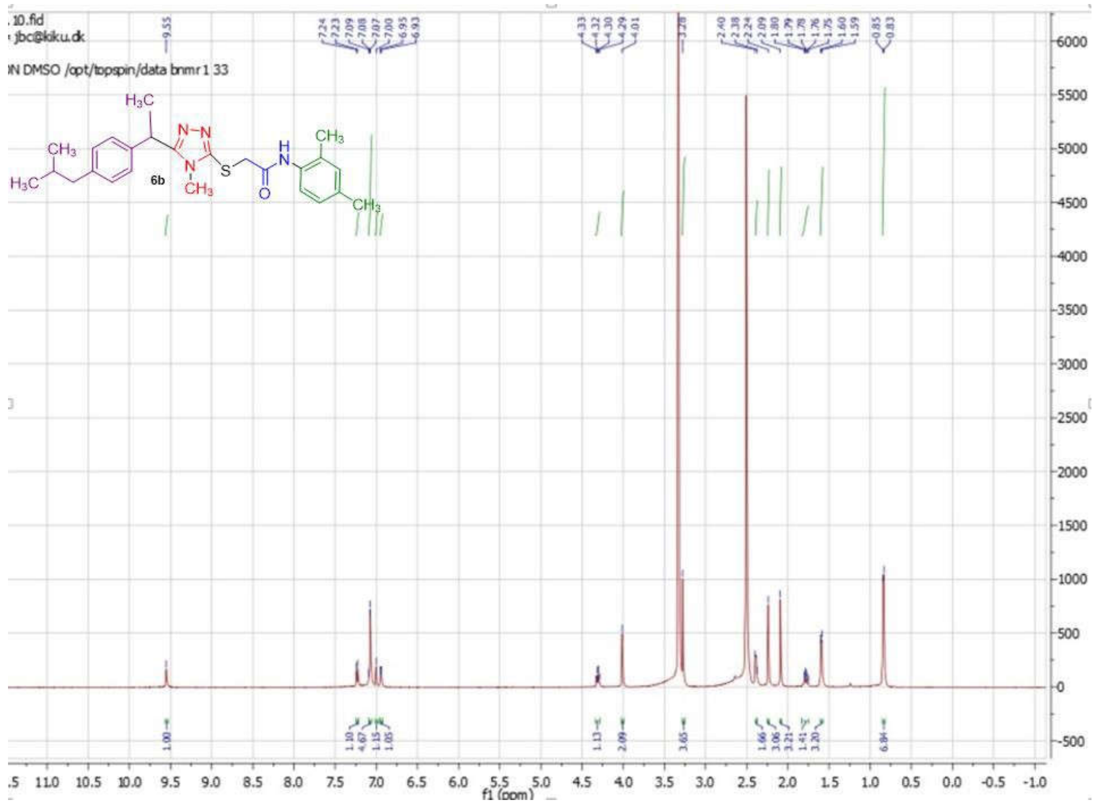

Figure S13. ^{1}H NMR spectrum of compound **6b** (Full spectrum)

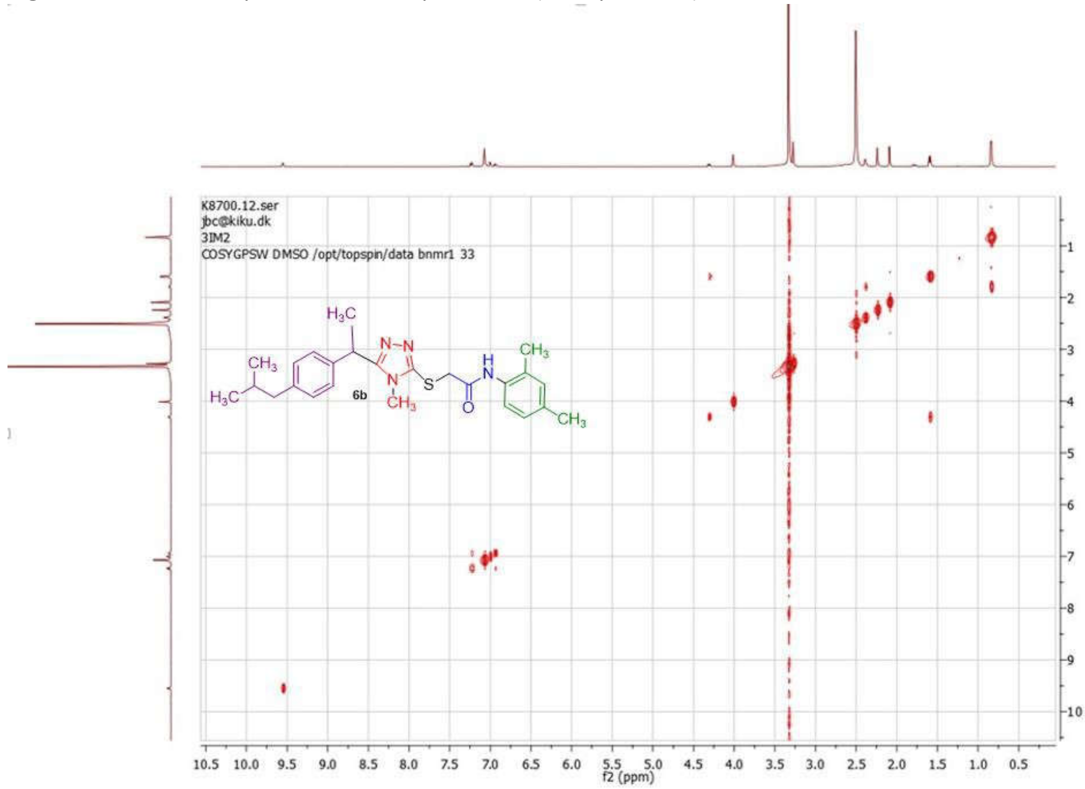

Figure S14. COSY- ^{1}H NMR spectrum of compound **6b**

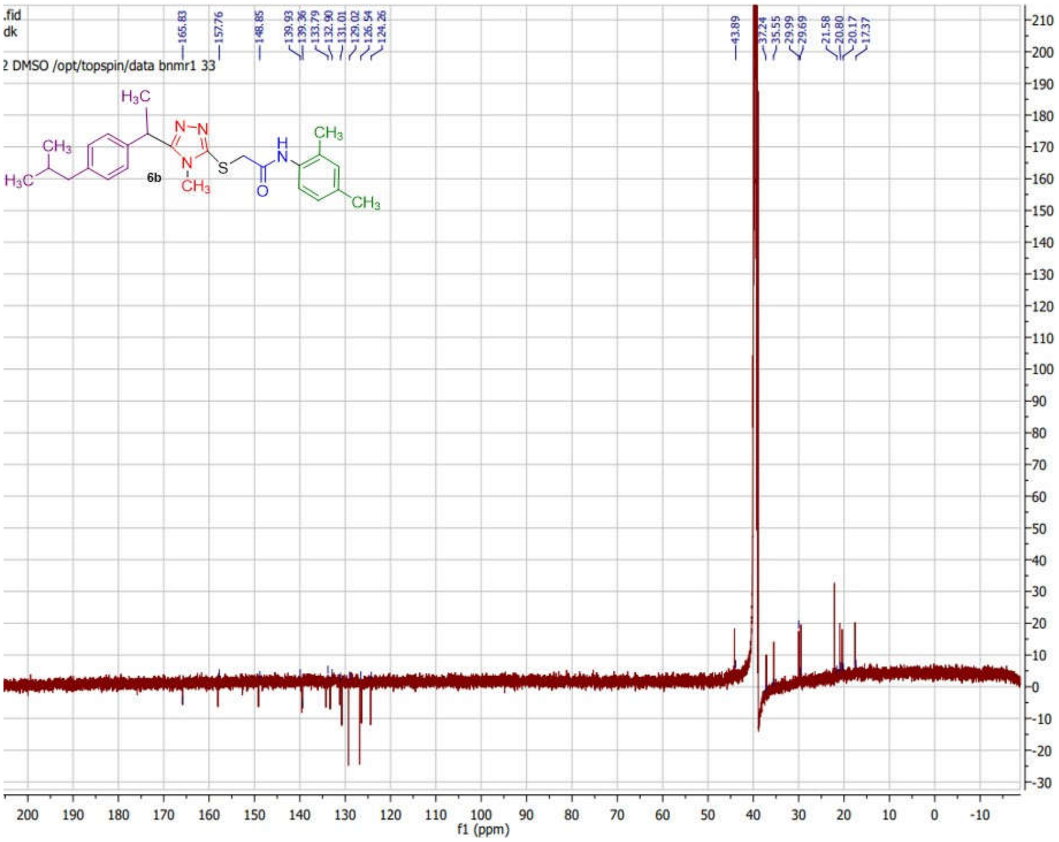

Figure S15. ^{13}C NMR spectrum of compound **6b** (Full spectrum)

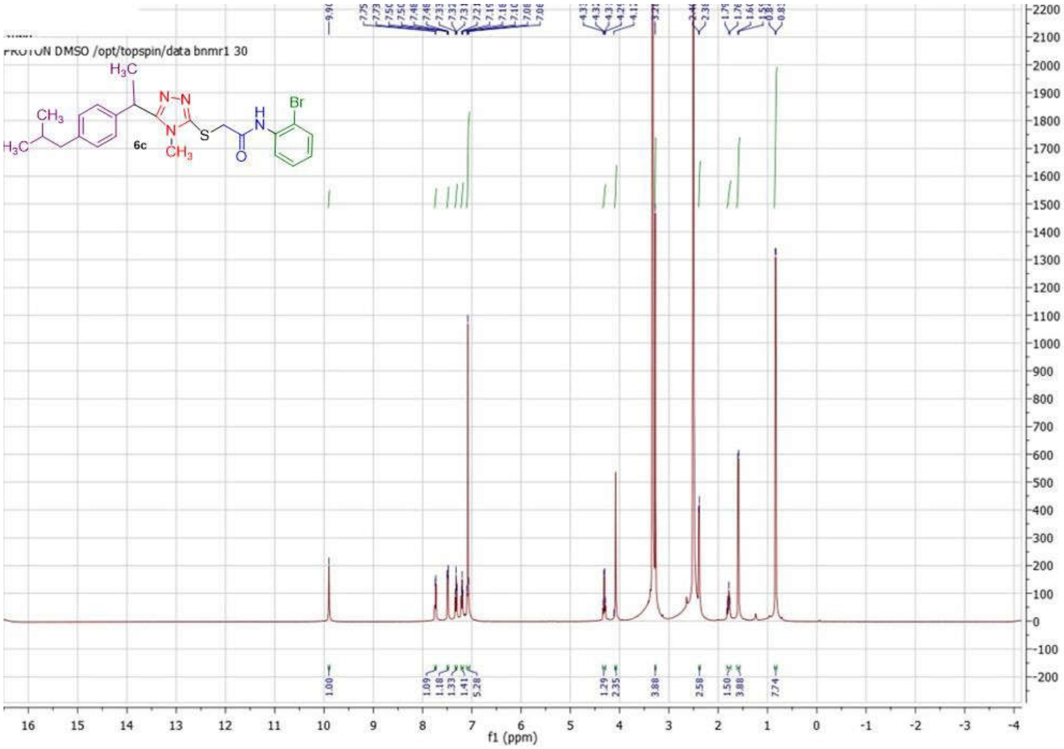

Figure S16. ^{1}H NMR spectrum of compound **6c** (Full spectrum)

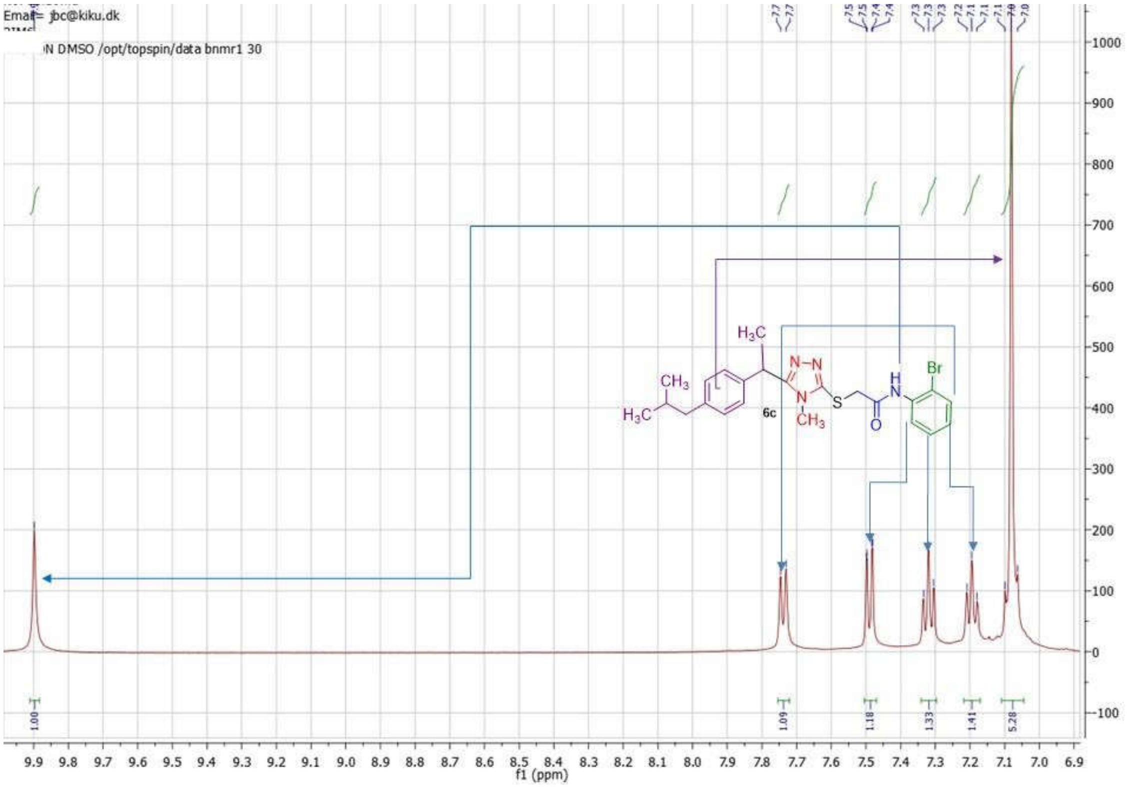

Figure S17. ^{1}H NMR spectrum of compound **6c** (aromatic region)

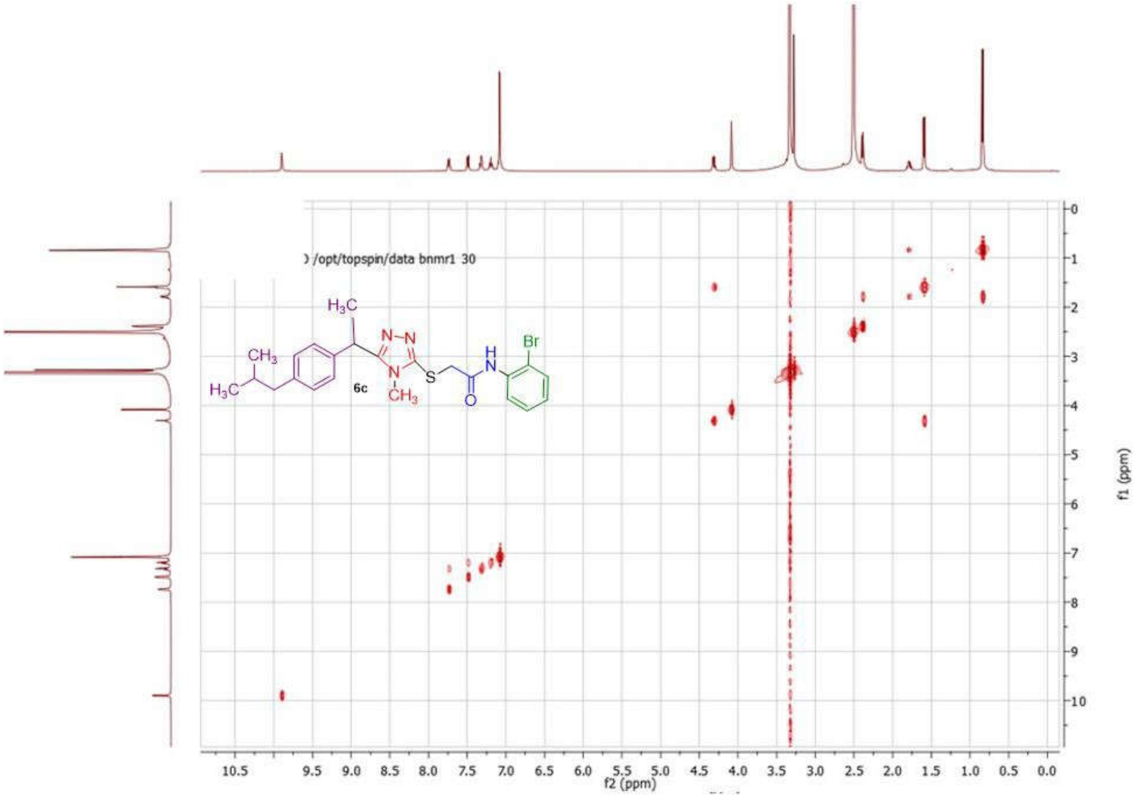

Figure S18. COSY-^{1}H NMR spectrum of compound **6c**

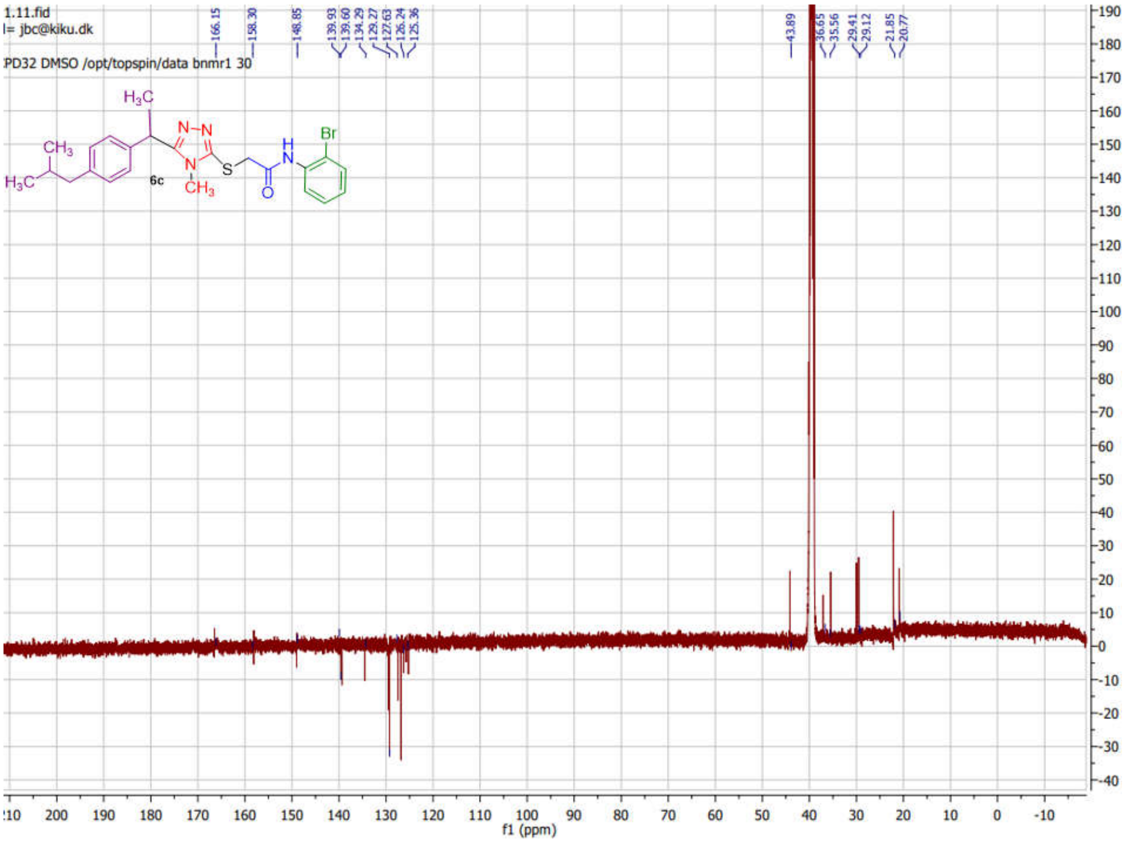

Figure S19. ^{13}C NMR spectrum of compound **6c** (Full spectrum)

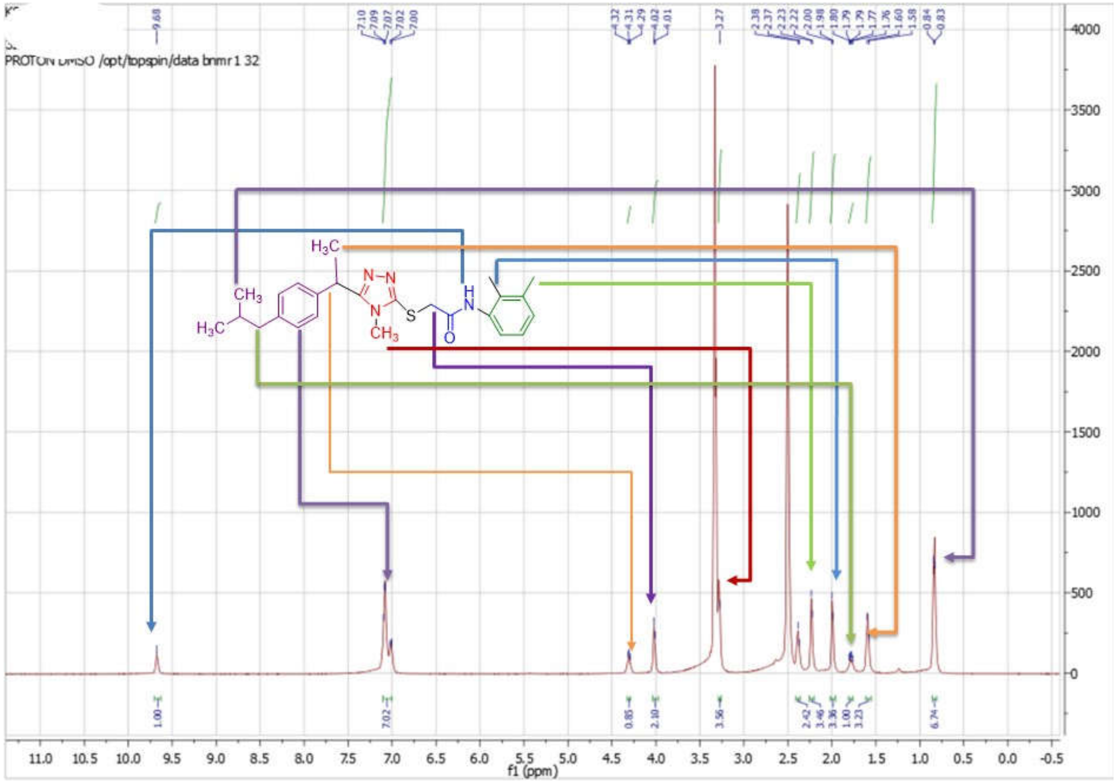

Figure S20. ^{1}H NMR spectrum of compound **6d** (Full spectrum)

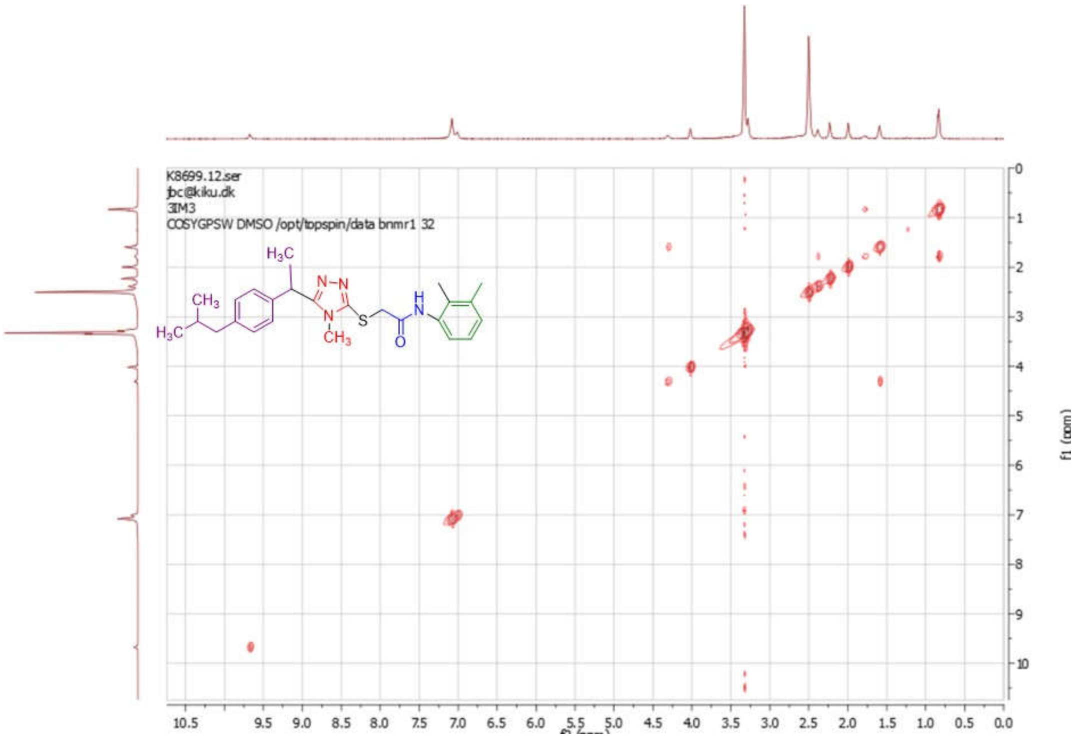

Figure S21. COSY ^{1}H NMR spectrum of compound **6d**

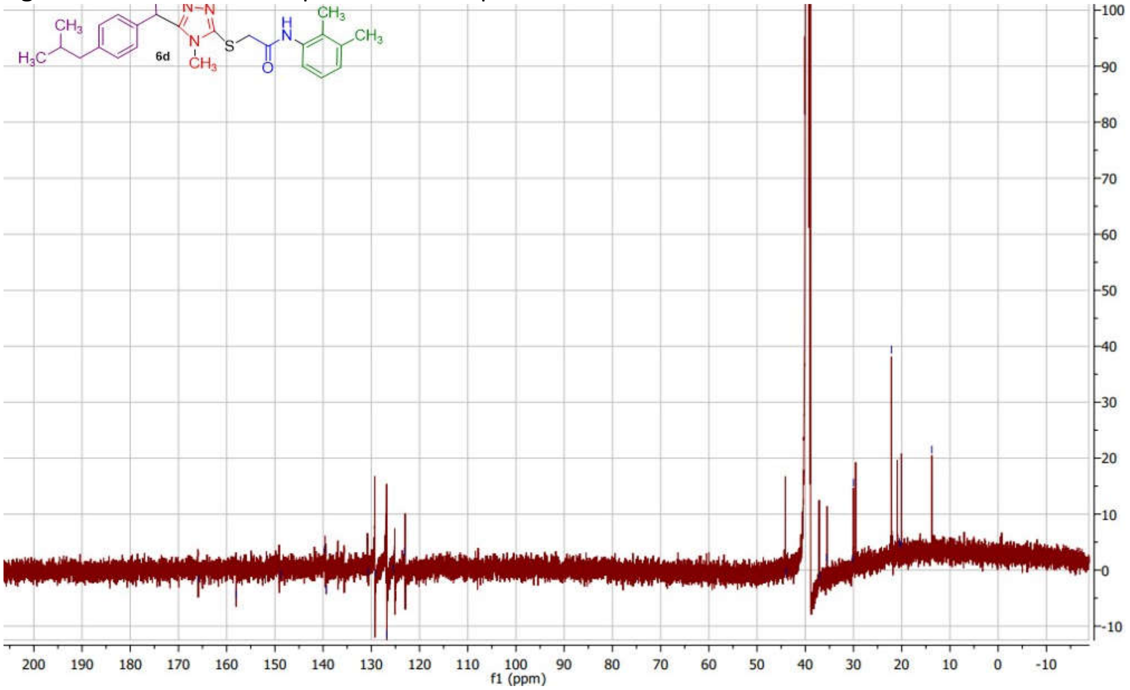

Figure S22. ^{13}C NMR spectrum of compound **6d** (Full spectrum)

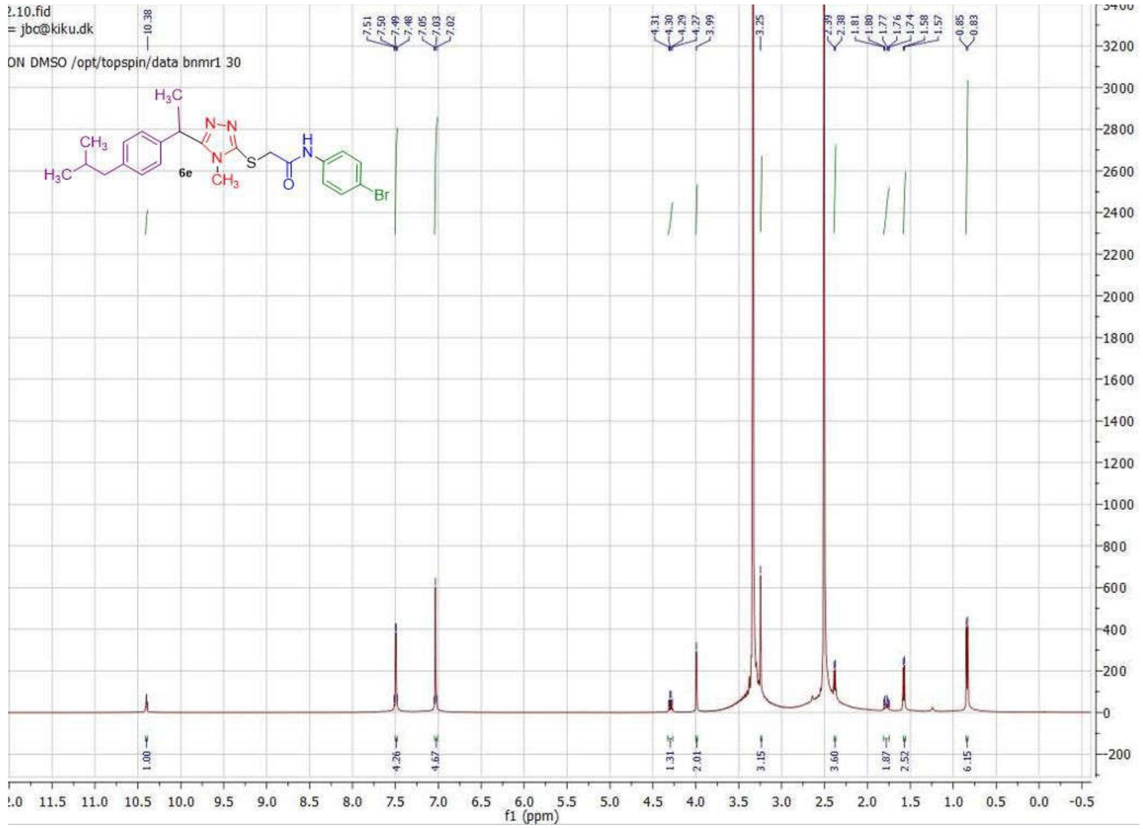

Figure S23. ^{1}H NMR spectrum of compound **6e** (Full)

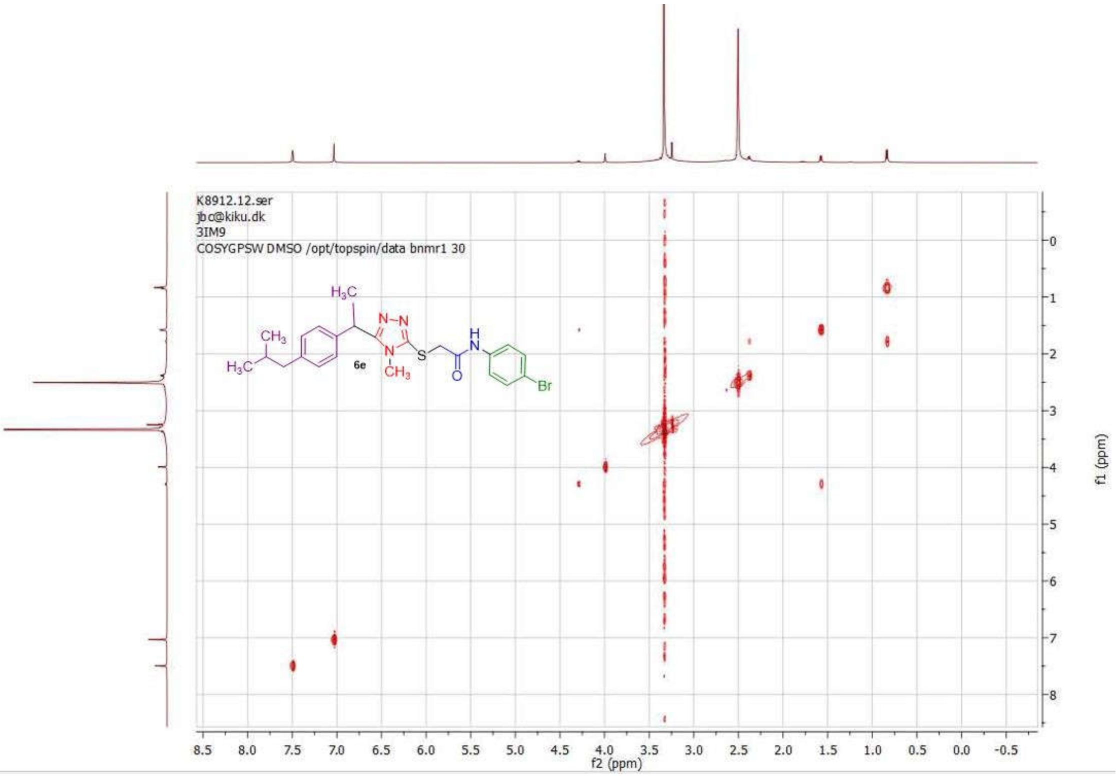

Figure S24. COSY ^{1}H NMR spectrum of compound **6e**

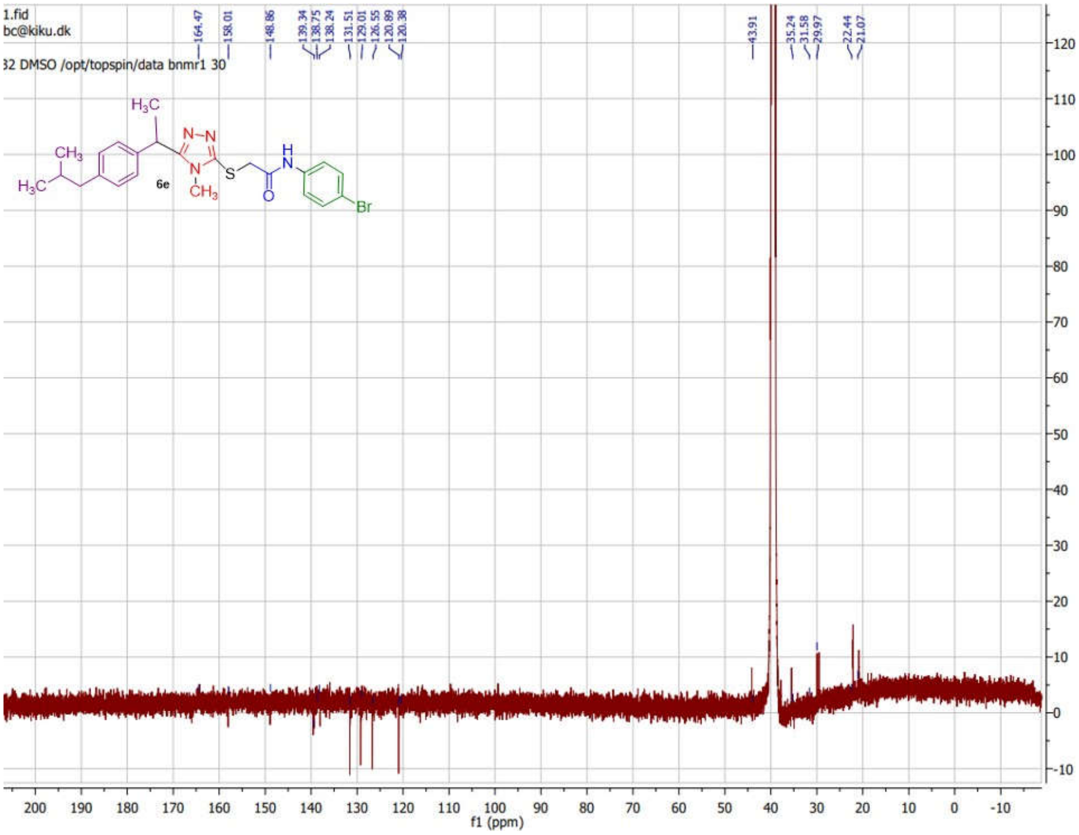

Figure S25. ^{13}C NMR spectrum of compound **6e** (Full spectrum)

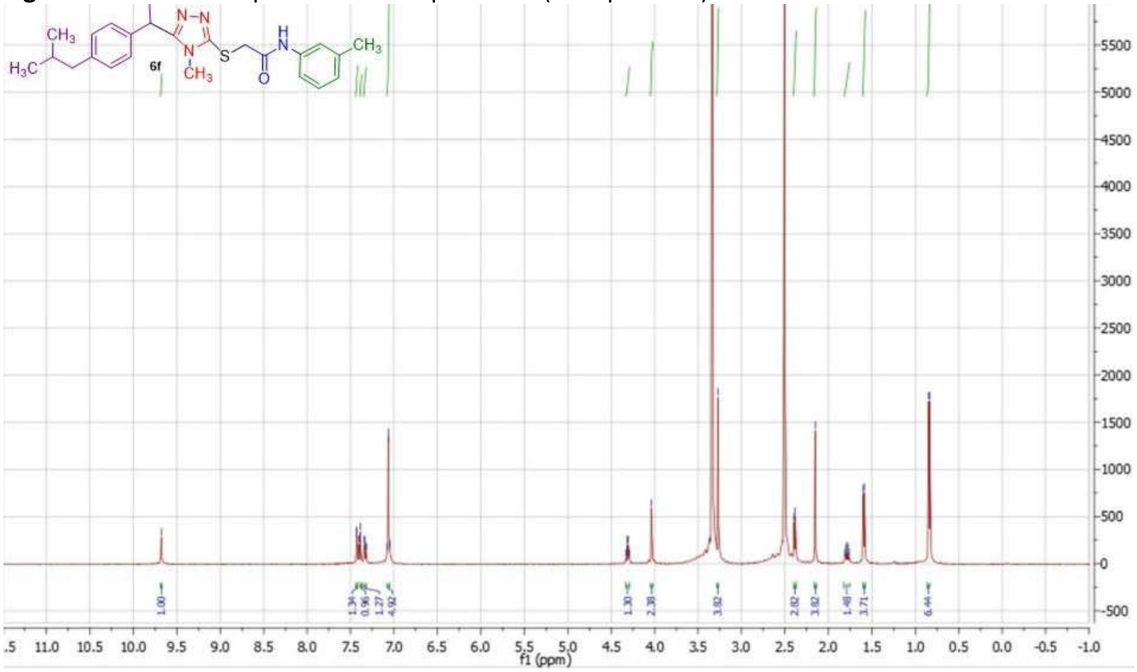

Figure S26. ^{1}H NMR spectrum of compound **6f** (Full Spectrum)

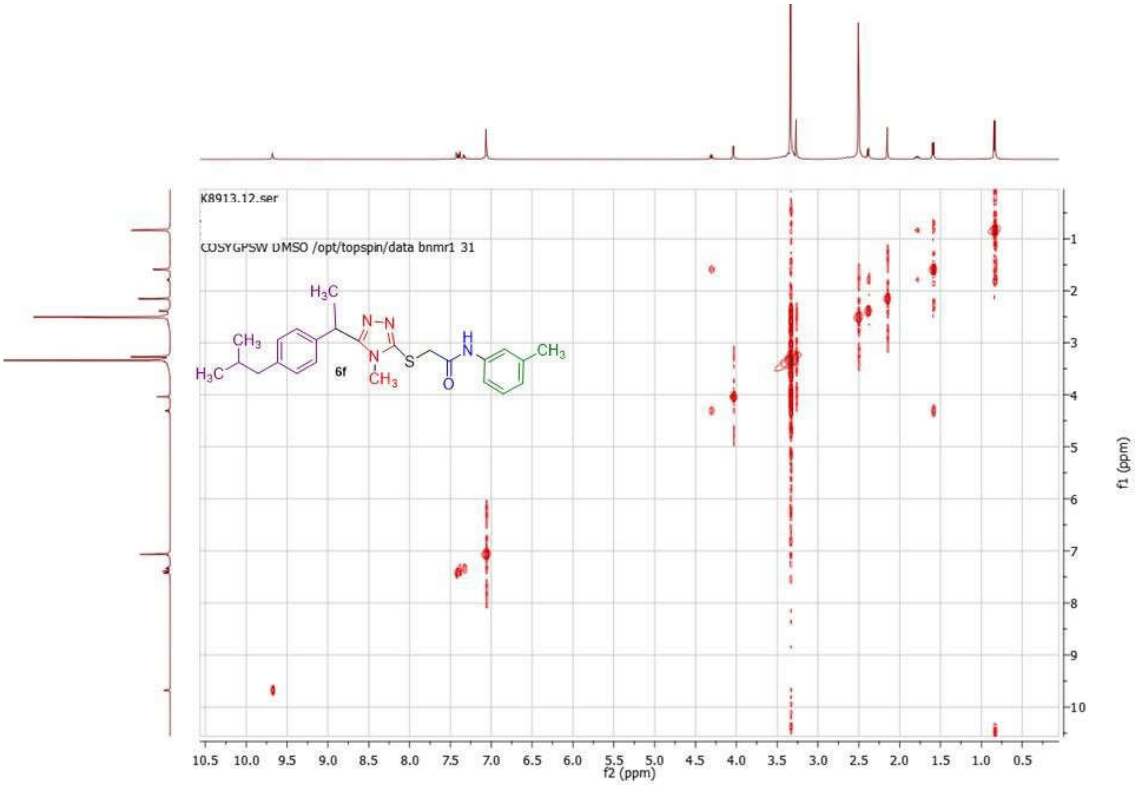

Figure S27. COSY ^{1}H NMR spectrum of compound **6f**

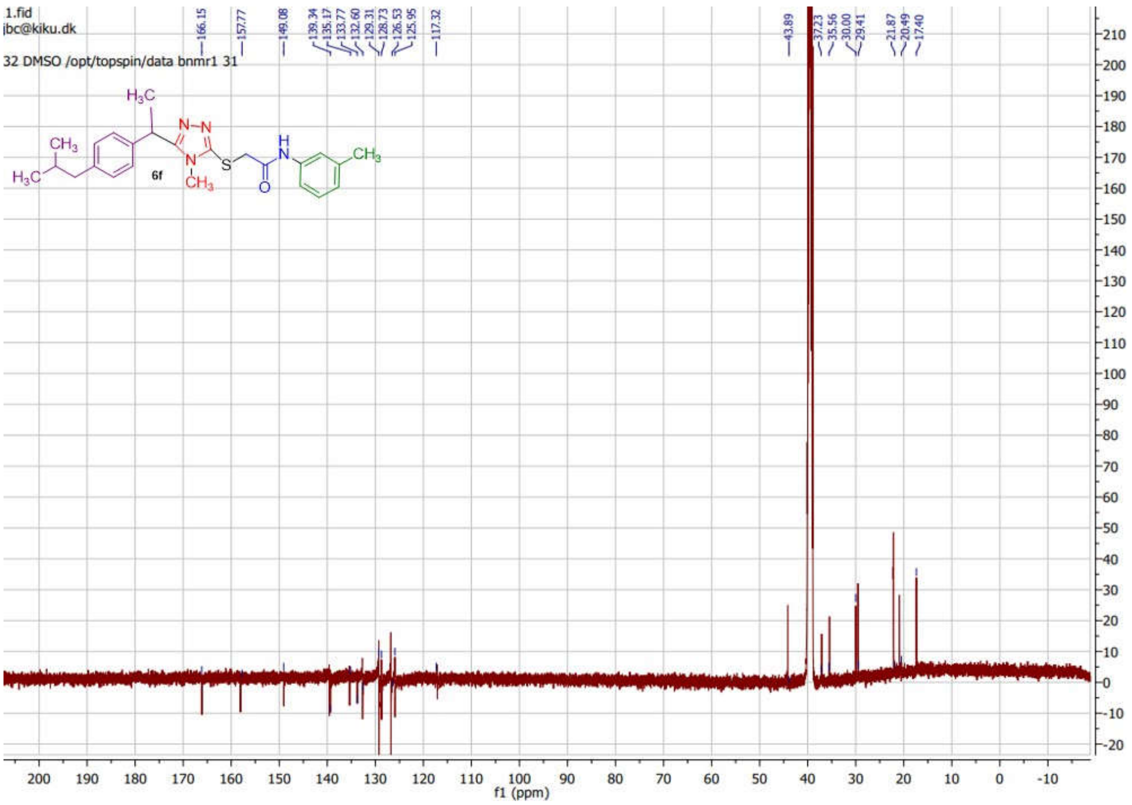

Figure S28. ^{13}C NMR spectrum of compound **6f**

Generic Display Report

**Analysis Info**		Acquisition Date	7/6/2022 7:10:30 AM
Analysis Name	S:\fticr0\rod\analyser-rutine\acq21198_0_H1_000001.d		
Method	10800	Operator	
Sample Name	IM11	Instrument	solariX XR
Comment			

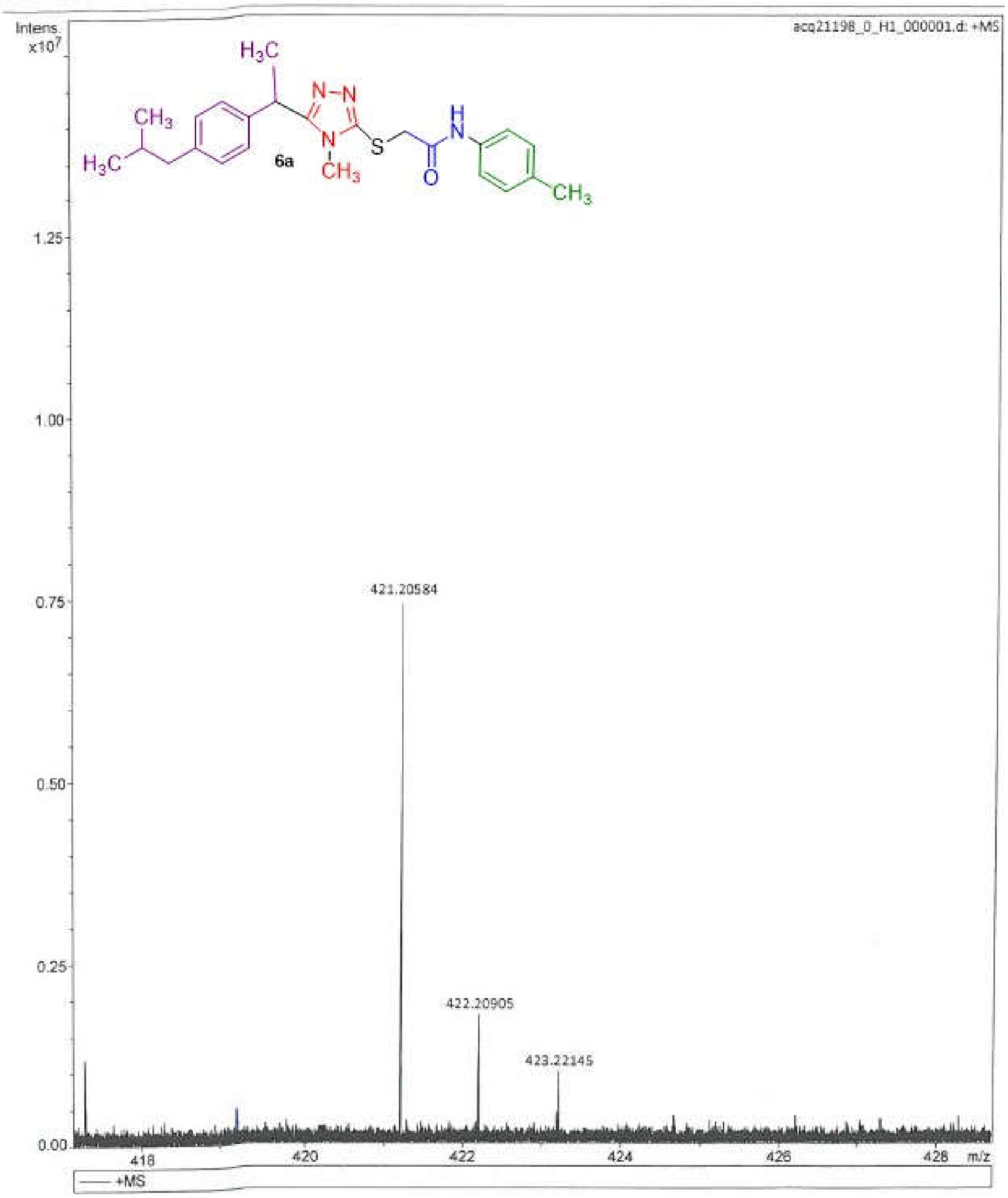

Figure S29. Mass Spectrum of compound **6a**

Generic Display Report

Analysis Info

Analysis Name	S:\fticr0\rod\analyser-rutine\acq21172_0_C1_000001.d	Acquisition Date	7/4/2022 8:31:12 AM
Method	ESI Pos 200-1200 4M Tuned mz 702_86 Calibrated	Operator	
Sample Name	27-6-2022	Instrument	solariX XR
Comment			

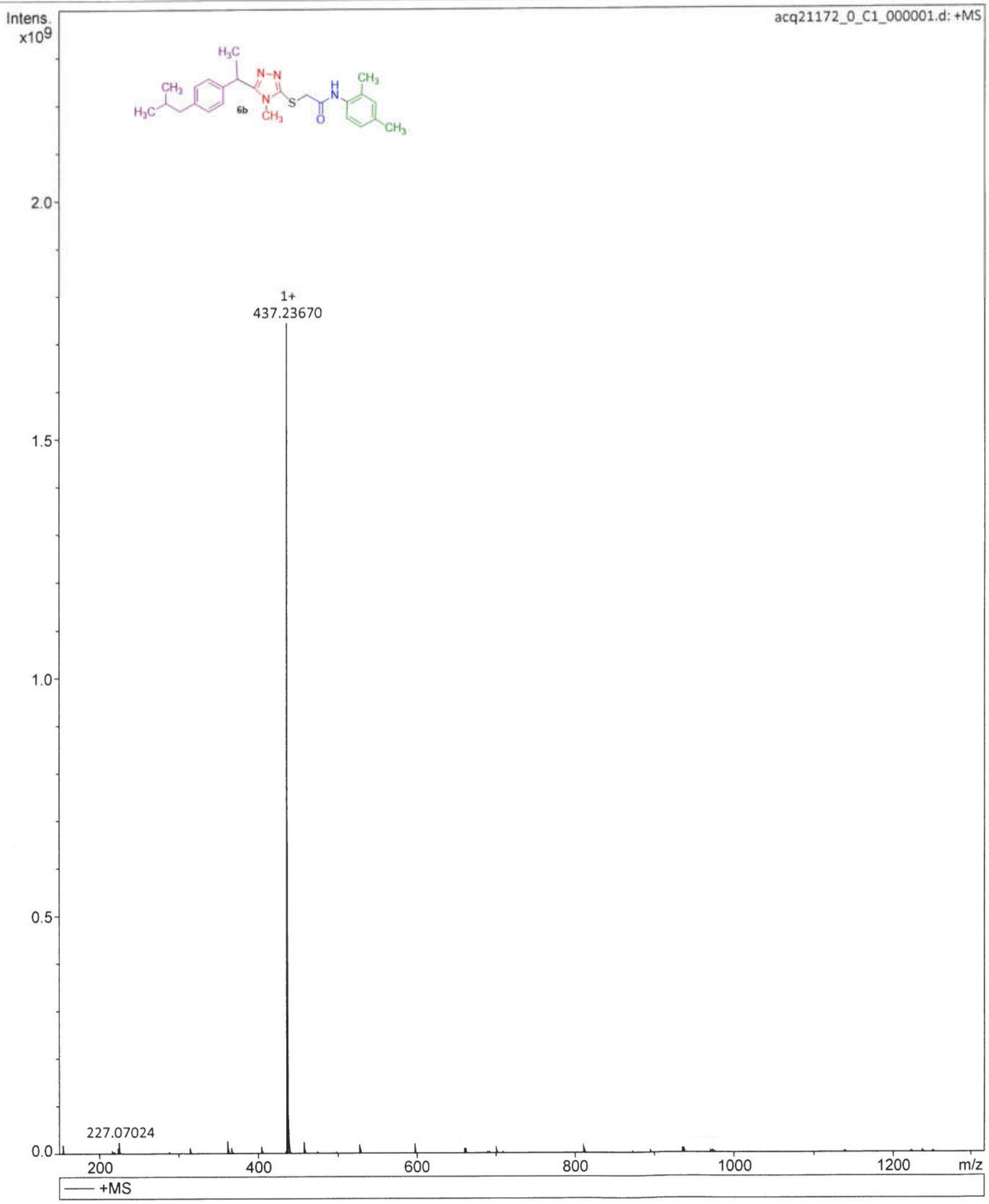

Figure S30. Mass Spectrum of compound **6b**

Generic Display Report

nalysis Info

nalysis Name	S:\fticr0\rod\analyser-rutine\acq21201_0_K1_000001.d	Acquisition Date	7/6/2022
lethod	10800	Operator	
ample Name	IM	Instrument	solariX XR
omment			

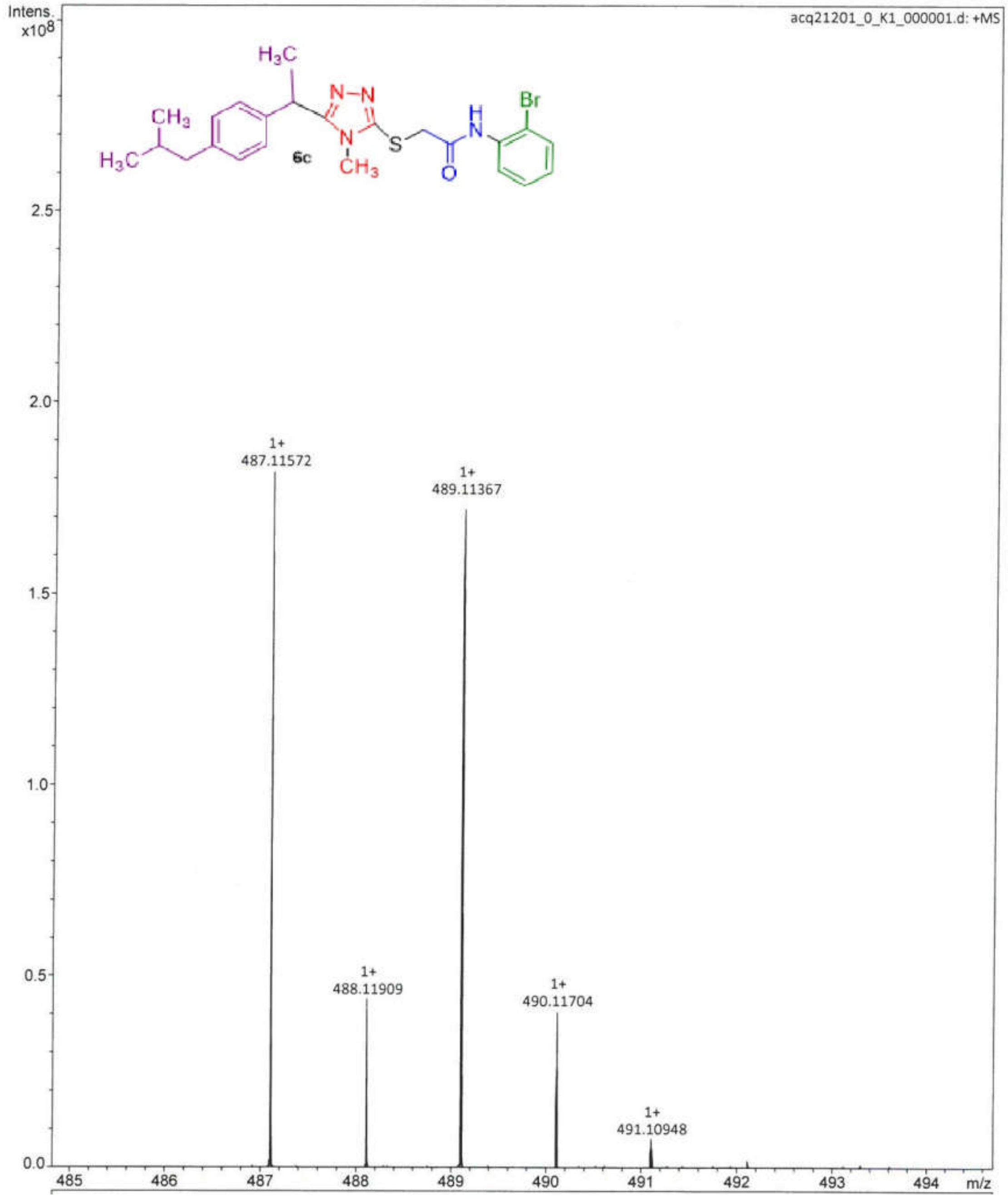

Figure S31. Mass Spectrum of compound **6c**

Generic Display Report

**Analysis Info**		Acquisition Date	7/4/2022 8:32:12 AM
Analysis Name	S:\fticr0\rod\analyser-rutine\acq21173_0_D1_000001.d		
Method	ESI Pos 200-1200 4M Tuned mz 702_86 Calibrated	Operator	
Sample Name	27-6-2022 IM3	Instrument	solariX XR
Comment			

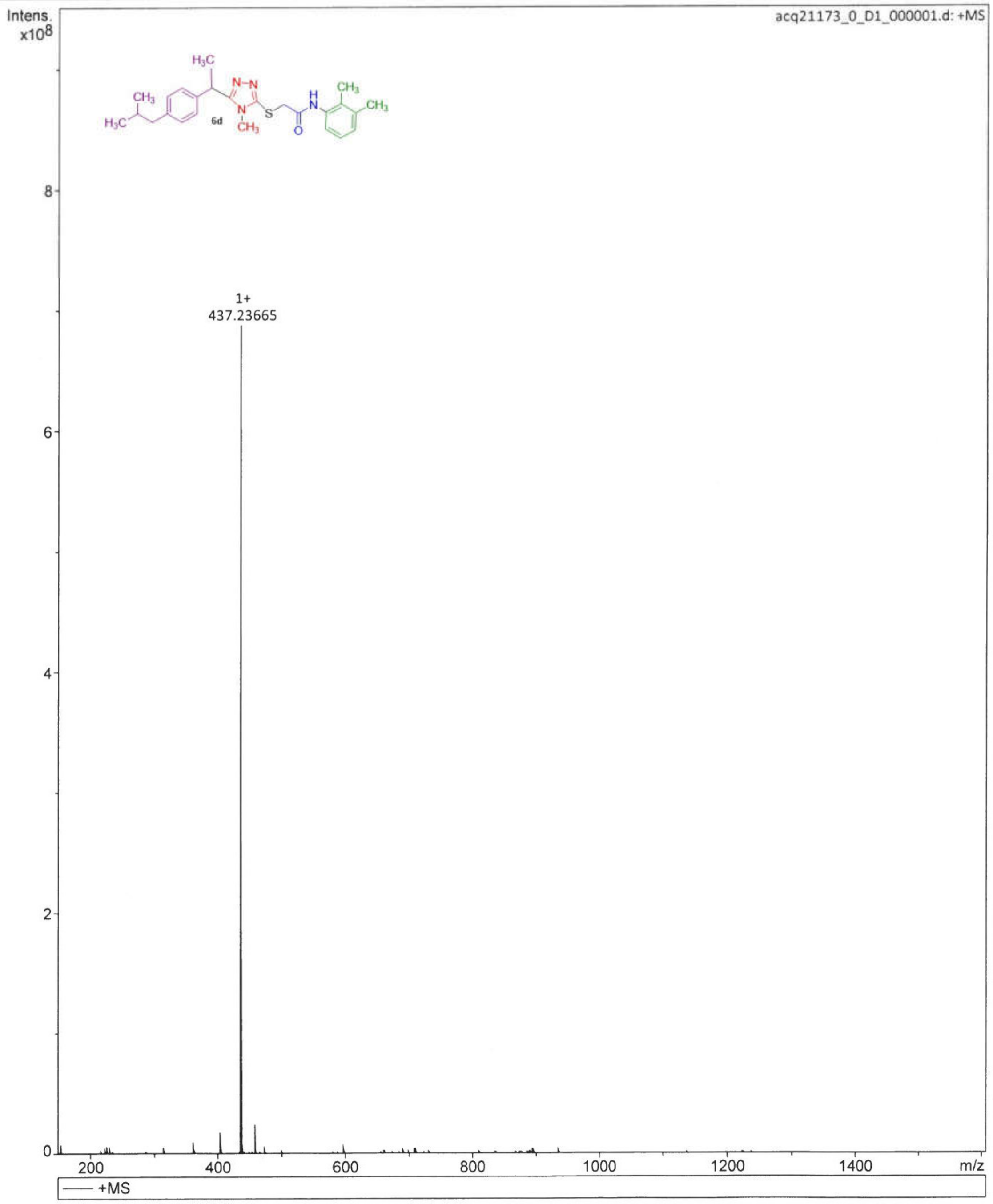

Figure S32. Mass Spectrum of compound **6d**

Generic Display Report

Analysis Info

Analysis Name S:\fticr0\rod\analyser-rutine\acq21201__O_K1_000001.d
Method 10800
Sample Name IM[illegible]
Comment

Acquisition Date 7/6/2022
Operator
Instrument solariX XR

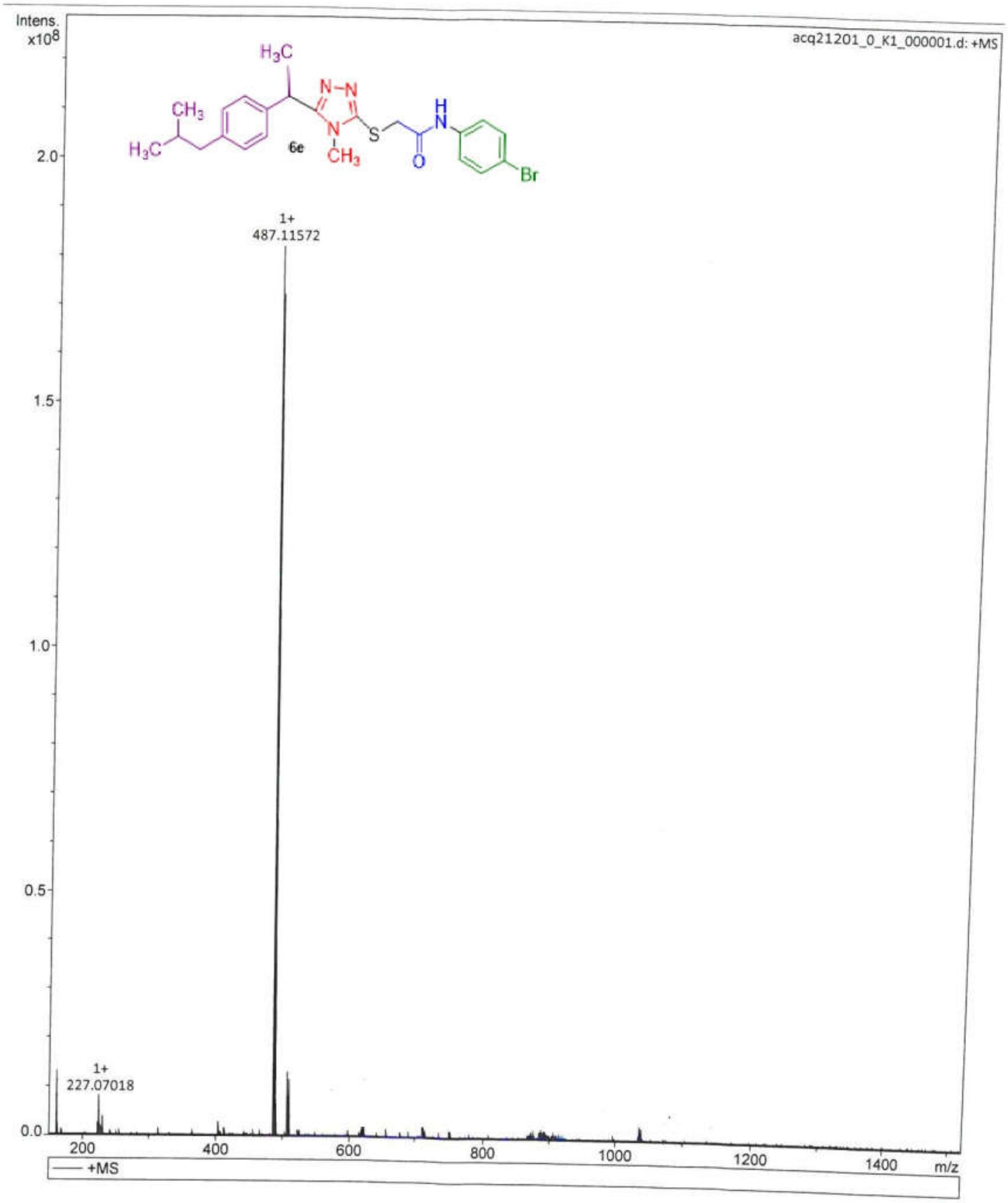

ruker Compass DataAnalysis 5.0 printed: 7/6/2022 7:14:47 AM by: demo [illegible]

Figure S33. Mass Spectrum of compound **6e**

Generic Display Report

Analysis Info

Analysis Name S:\fticr0\rod\analyser-rutine\acq21202_0_L1_000001.d
Method 10800
Sample Name IM10
Comment

Acquisition Date 7/6/2022
Operator
Instrument solariX XR

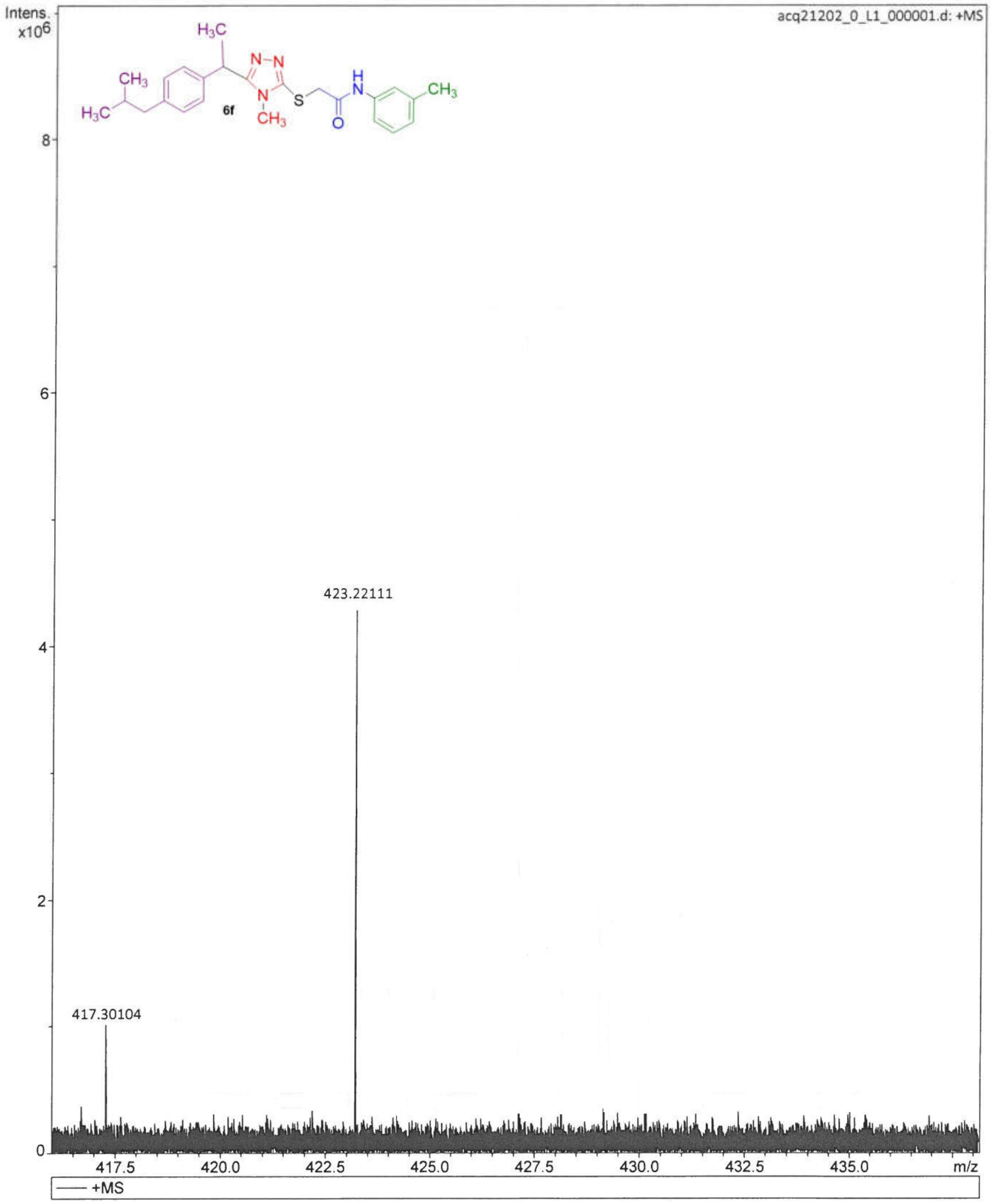

Figure S34. Mass Spectrum of compound **6f**
